# COVID-19 vaccine hesitancy and its drivers: An empirical study of the vaccine hesitant group in Malaysia

**DOI:** 10.1371/journal.pone.0282520

**Published:** 2023-03-15

**Authors:** Santha Vaithilingam, Li-Ann Hwang, Mahendhiran Nair, Jason Wei Jian Ng, Pervaiz Ahmed, Kamarul Imran Musa

**Affiliations:** 1 Sunway Institute for Global Strategy and Competitiveness, Sunway University, Selangor, Malaysia; 2 Department of Econometrics and Business Statistics, School of Business, Monash University, Selangor, Malaysia; 3 Department of Pure and Applied Mathematics, School of Mathematical Sciences, Sunway University, Selangor, Malaysia; 4 Department of Community Medicine, School of Medical Sciences, Universiti Sains Malaysia, Kelantan, Malaysia; King Abdulaziz University Faculty of Medicine, SAUDI ARABIA

## Abstract

**Background:**

Sporadic outbreaks of COVID-19 remain a threat to public healthcare, especially if vaccination levels do not improve. As Malaysia begins its transition into the endemic phase, it is essential to identify the key determinants of COVID-19 vaccination intention amongst the pockets of the population who are still hesitant. Therefore, focusing on a sample of individuals who did not register for the COVID-19 vaccination, the current study integrated two widely used frameworks in the public health domain—the health belief model (HBM) and the theory of reasoned action (TRA)—to examine the inter-relationships of the predictors of vaccination intention amongst these individuals.

**Methodology:**

Primary data from 117 respondents who did not register for the COVID-19 vaccination were collected using self-administered questionnaires to capture predictors of vaccination intention amongst individuals in a Malaysian context. The partial least squares structural equation modeling (PLS-SEM) technique was used to analyze the data.

**Results:**

Subjective norms and attitude play key mediating roles between the HBM factors and vaccination intention amongst the unregistered respondents. In particular, subjective norms mediate the relationship between cues to action and vaccination intention, highlighting the significance of important others to influence unregistered individuals who are already exposed to information from mass media and interpersonal discussions regarding vaccines. Trust, perceived susceptibility, and perceived benefits indirectly influence vaccination intention through attitude, indicating that one’s attitude is vital in promoting behavioral change.

**Conclusion:**

This study showed that the behavioral factors could help understand the reasons for vaccine refusal or acceptance, and shape and improve health interventions, particularly among the vaccine-hesitant group in a developing country. Therefore, policymakers and key stakeholders can develop effective strategies or interventions to encourage vaccination amongst the unvaccinated for future health pandemics by targeting subjective norms and attitude.

## Introduction

On March 11, 2020, the World Health Organization (WHO) declared the coronavirus disease (COVID-19) a global pandemic. Since then, the COVID-19 pandemic has caused unprecedented public health threats that continue to linger globally after two years. While some countries have transitioned to the endemic phase, the WHO cautions that the pandemic is far from over and could continue to spark global outbreaks if vaccination levels decline [1, 2]. Since the authorization of COVID-19 vaccines in late 2020, vaccine uptake has been the focus for countries to achieve herd immunity [3, 4].

From the public health perspective, widespread vaccination is regarded as the most effective measure to combat infectious disease outbreaks [5–7]. As of 25 October 2022, Malaysia has reported nearly 4.9 million COVID-19 cases, with 84.3% of the total population vaccinated against COVID-19. By global standards, Malaysia’s vaccination rate is relatively high compared to other countries. Nevertheless, vaccine hesitancy, defined by the WHO as a “delay in acceptance or refusal of vaccines despite availability of vaccine services” [8], remains a challenge for pockets of the population as the country transitions to the endemic phase [2, 9]. For example, those above 80 years old (80.9%) and six out of the 16 states in Malaysia (Kedah (77.4%), Kelantan (65.0%), Pahang (76.0%), Perak (80.7%), Sabah (68.2%), Terengganu (73.4%)) have a primary vaccination coverage lower than the national average as of 25 October 2022 [10].

Notwithstanding Malaysia’s relatively high overall vaccination coverage, vaccine hesitancy remains a valid concern for three primary reasons. First, the mortality rate among the unvaccinated is high. Out of the country’s 36,452 COVID-19 recorded deaths as of 25 October 2022, 61.2% were unvaccinated [10]. Second, improving vaccination coverage not only impacts mortality rates but also correlates with positive public and individual health outcomes, such as lowering the transmission rate of COVID-19 and reducing the severity and complications arising from COVID-19 infections [11, 12]. Third, the effectiveness of vaccine coverage is jeopardized by the current need for vaccine boosters to enhance or restore protection that might have waned over time after the primary rounds of vaccination [13–15]. Therefore, at least in the foreseeable future, continued vaccination will be required. Hence, obtaining behavioral insights on why an attitude of vaccine hesitancy persists is crucial in effectively managing the pandemic [16]. This is especially relevant in light of the comments by Malaysia’s Minister of Health, who has affirmed that employing behavioural science will be vital in combating subsequent spreads of COVID-19 [17].

Malaysia started its COVID-19 vaccination program on February 24, 2021. However, it is a requirement for residents who want to be vaccinated to register before vaccination. Hence, in contrast to most prior Malaysian research that has investigated the vaccination intention of whole populations without regard for their registration status [18–20], this study narrows into the vaccination intention of the unregistered population segment, which represents the core of the vaccine-hesitant. Therefore, this unique context enables the understanding of the underlying factors and how these factors, defined as situational opportunities or constraints, play a crucial role in shaping the behavior of this group of people [21, 22]. In particular, we focus on identifying the behavioral factors that can convince, incentivize, and encourage this vaccine-hesitant group to receive the vaccine. In doing so, healthcare providers and the government can implement effective strategies for interventions in the future for this group.

Against this backdrop, the primary objective of this study is to examine the key determinants of COVID-19 vaccination intention amongst individuals who did not register for vaccination. To do this, this study integrates two widely used theoretical frameworks—the health belief model (HBM) [23] and the theory of reasoned action (TRA) [24] to predict vaccination intention. This contrasts with prior studies that only employ the HBM when investigating the acceptance of COVID-19 vaccines [7, 18]. A notable exception is a recent study by Ng et al. [20], which also adopted both the HBM and TRA to predict vaccination intention. However, Ng et al. [20] employed regression-type modelling methods to the combined sample of registered and unregistered individuals, while this study uses structural equation modelling (SEM) to examine the inter-relationships between the factors among the unregistered individuals. SEM allows latent variables to be endogenous and, at the same time, exogenous. Compared to regression models, SEM allows the evaluation of multiple independent and dependent variables simultaneously [25].

The survey administered for this study was conducted in early June 2021, when Malaysia’s daily reported COVID-19 cases were at their highest and at a time when vaccines were more widely available. By this time, the population also has had a three-month window to register for vaccination via a telephone call or the government-developed mobile phone application, MySejahtera. Therefore, this study differs from earlier Malaysian studies conducted when the COVID-19 infection was not as severe and before vaccines were readily available [7, 26]. Based on these developments, this study provides an unparalleled understanding of vaccination intention amongst unregistered participants who represent the core group of the vaccine-hesitant.

Given that vaccinations play a vital role in controlling the spread of not only COVID-19 [27] but also other infectious diseases, it becomes increasingly important to identify why specific individuals are still hesitant about getting vaccinated. Doing so would allow policymakers to develop targeted programs, campaigns, or policies geared toward individuals who are hesitant to be vaccinated. The findings from this study would also help inform public administration practitioners and health authorities about managing future pandemics.

## Conceptual framework

**Fig 1:**
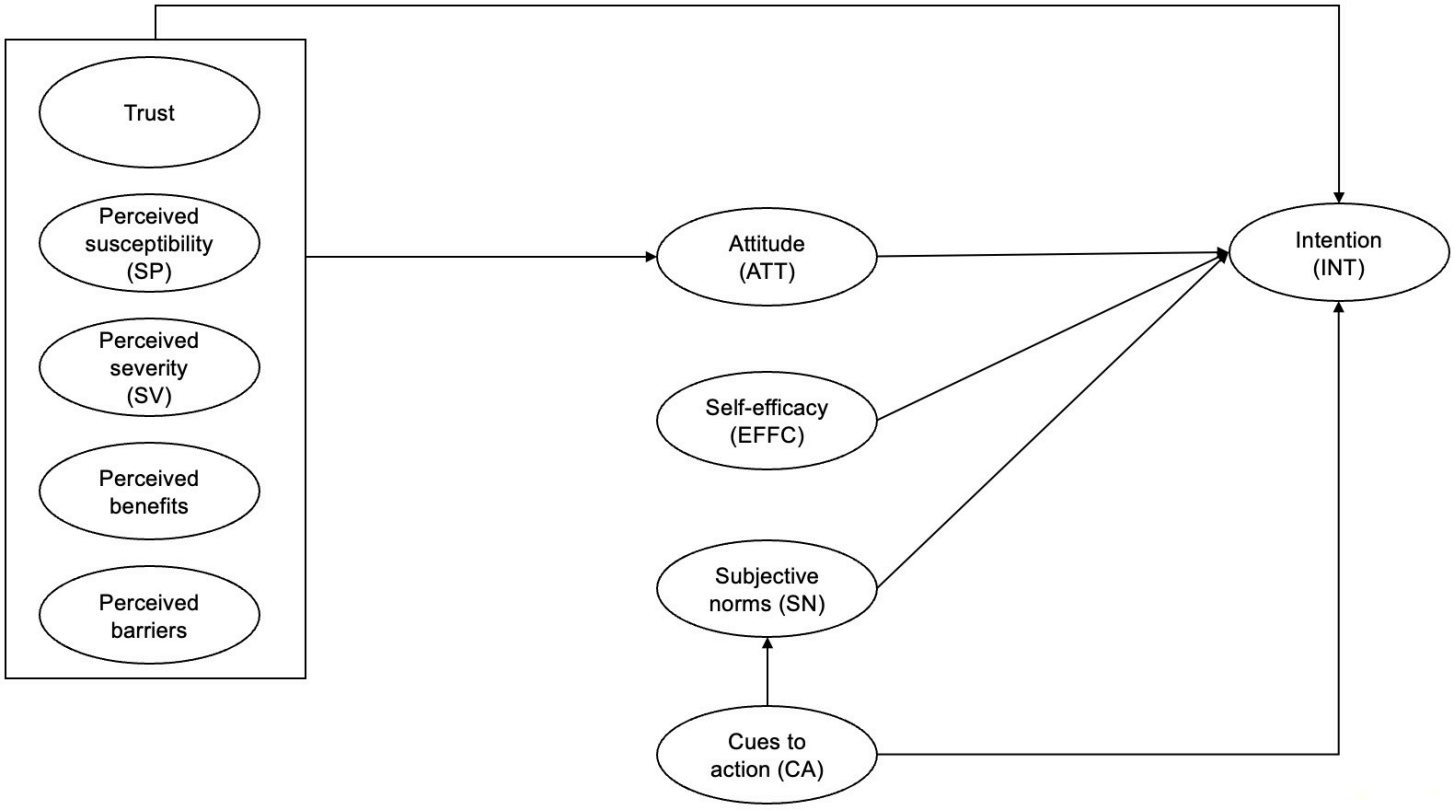
Conceptual framework.

In this study, we integrate the HBM and TRA to examine vaccination intention amongst individuals who did not register for the COVID-19 vaccine (refer to Fig 1). We also included trust in this integrated model, which encompasses trust in the vaccine process, information sources, and the government [28–30].

The HBM, largely used to predict health behaviors, comprises six key domains, namely, perceived susceptibility (SP), perceived severity (SV), perceived benefits, perceived barriers, cues to action (CA), and self-efficacy (EFFC). Applying these factors to the COVID-19 context, the HBM postulates that individuals are more likely to get vaccinated based on their perceptions of the benefits of and the barriers to vaccination, and their perceptions of the susceptibility to and severity of the virus. Cues to action refer to where an individual acts upon vaccination behavior based on stimuli such as interpersonal discussion and information from mass media.

The HBM is a value-expectancy theory which assumes that an individual’s behavior is guided by expectations of the outcomes of adopting new health practices [31]. However, key social cognitive variables that have been found to be highly predictive of behaviour in other models are not in the HBM [32]. Such variables include the intention to perform a behaviour and social pressure. Therefore, we integrate the HBM with the TRA in this study.

The TRA provides an understanding of human behavior in various contexts. This theory hypothesizes that an individual’s intention to vaccinate depends on two factors—attitude and subjective norms. Attitude is integral to influencing behavioral change, while subjective norms affect individuals’ decision-making based on the perception of important others [33, 34]. Therefore, integrating the TRA into the conceptual framework will provide insights into the influence of subjective norms and attitude on vaccination intention amongst the unregistered population. This could subsequently allow policymakers to design targeted interventions and communications to promote vaccine uptake for this population group.

## Methodology

### Data collection

Data was collected by an international marketing consulting firm in Malaysia using self-administered questionnaires via its online panel from 11 to 20 June 2021. The consulting firm’s online panel has a reach of 32,000 panelists throughout the country. The panelists’ age group, gender and region are similar to Malaysia’s population census distribution. A representative stratified random sample of respondents was selected and contacted via email.

To assuage potential concerns about data quality assurance due to third-party data collection, we selected the consulting firm that has an established track record of conducting and reporting national-level surveys. They also actively maintain their online panel to ensure a strong sample quality commitment. For example, they have safeguards such as name/address validation, country validation via Geo-IP, anonymous proxy and 5-minute email detection, detection of data anomalies and patterns, and matching respondents against a blacklist. They also monitor survey-taking behaviors such as speeding and straight-lining.

In this study, we focus on the 117 respondents aged 18 years and above who indicated that they did not register for the COVID-19 vaccine. As Malaysia was under a nationwide lockdown at the time of data collection, the consulting firm could not collect data from the rural population. Therefore, the sample in this study consists of a more urban, connected population.

Of the 117 respondents, 59% were males, while 48% were females. 60% of the respondents were below 40 years old, while the remaining were aged between 41 to 80 years. Regarding the highest level of education attained, 38% attained tertiary education, and the remaining 62% had lower educational attainment.

### Measures

The questionnaire was developed based on validated instruments from prior studies and a few self-developed items to capture perceptions specific to the Malaysian context. This study’s primary measure of interest is an individual’s intention to receive COVID-19 vaccination.

We included six constructs from the HBM to measure respondents’ perceived susceptibility, perceived severity, perceived benefits, perceived barriers, cues to action, and self-efficacy. Items measuring these constructs were obtained from several sources [35–37]. In the context of this study, perceived susceptibility refers to an individual’s assessment of the risk of being infected by COVID-19, whereas perceived severity is defined as one’s belief about the seriousness of COVID-19 infections [23]. Perceived benefits refer to the positive outcomes of getting vaccinated, while perceived barriers refer to one’s assessment of the influences that hinder or discourage vaccination [23]. This study operationalized perceived barriers to comprise three distinct barriers—access/logistics, registration, and religion. Cues to action are strategies to activate readiness leading to the performance of the behavior [23] and include two dimensions: media attention and interpersonal discussion. Self-efficacy, in this study, refers to an individual’s belief of being able to get the COVID-19 vaccine [38]. That is, individuals who possess the ability and knowledge to obtain COVID-19 vaccines are more likely to act on the behavior.

As we integrated the TRA into the conceptual framework, we included constructs from the TRA—attitude and subjective norms. The items used to measure these constructs were adapted from Chu and Liu [36] and Yang [37]. We defined attitude as an individual’s evaluative affect on getting the COVID-19 vaccine. It is a function of one’s salient beliefs about the outcomes of vaccination and the evaluation of those outcomes [39]. Subjective norms refer to an individual’s perception that most people who are important to them think they should or should not get the COVID-19 vaccine [33]. Essentially, subjective norms imply that although an individual may not find the behavior (i.e., getting the COVID-19 vaccine) or its result favorable, they may still decide to execute the behavior if important referents think that they should and they are motivated to comply with these referents [38, 40].

In addition to items from the HBM and TRA, we included trust, operationalized to consist of trust in the vaccine process, information sources, and the government [28–30].

All items were measured on a 5-point Likert scale and modified to suit the context of the study (see S1 Appendix for the complete list of items used in this study).

The questionnaire was developed in English and translated into Malay and Chinese by professional translators. The translated questionnaires were then back-translated to the original language to ensure that the original and translated questionnaires were similar to ensure reliability and validity. We also computed the content validity index (CVI) for each construct by inviting a panel of six experts to evaluate each item associated with each construct on a scale ranging from 1 to 4 (1 = not relevant, 2 = not important, 3 = relevant, 4 = important). Based on Lynn’s [41] recommendation, the mean CVI computed for each construct satisfies the threshold value, indicating adequate content validity for each construct.

### Data analysis

We used partial least squares structural equation modeling (PLS-SEM) to analyze the collected data. PLS-SEM was chosen due to its “causal-predictive” nature, allowing the model to obtain high predictive accuracy based on causal explanations [42–44]. The analysis was conducted using the SmartPLS 3.3.5 software [45].

### Ethics statement

We obtained ethics approval from the Monash University Human Ethics Committee (Project ID: 28249) prior to commencing data collection. Participants were required to provide written consent before the questionnaire was administered via the consulting firm’s online panel. Participant details such as names and other personal identifiers were de-identified.

## Results

The two-step procedure recommended by Anderson and Gerbing [46] was adopted to analyze the model. We first evaluated the measurement model for reliability, convergent validity, and discriminant validity. Once reliability and validity have been established, we assessed the structural model that represents the relationship between the constructs.

### Measurement model evaluation

All constructs in this study were measured using reflective indicators. Perceived barriers, cues to action, and trust were conceptualized as reflective-reflective higher-order constructs (HOCs). Including HOCs reduces the number of relationships in the structural model. Hence, the PLS-SEM becomes more parsimonious and easier to comprehend [47]. Table 1 shows the measurement model results. The Cronbach’s *α* and composite reliability (CR) are used to measure internal consistency reliability. Both measures for all constructs in Table 1 are above the suggested threshold of 0.7, indicating that internal consistency is achieved [42, 47]. Indicator reliability is evaluated through standardized outer loadings. Most of the outer loadings satisfy the 0.7 threshold. Those indicators loadings that are below 0.7 but above 0.4, are retained as they were relevant in measuring the construct [47, 48]. The AVE values are all above the suggested value of 0.5, indicating adequate convergent validity.

**Table 1:** Measurement model evaluation.

Construct	Indicators	Outer Loadings	Cronbach’s *α*	CR	AVE
ATT	ATT1	0.951	0.921	0.950	0.864
	ATT2	0.921			
	ATT3	0.916			
BENEFIT	CBENF1	0.857	0.925	0.941	0.728
	CBENF2	0.881			
	CBENF3	0.755			
	IBENF1	0.861			
	IBENF2	0.893			
	IBENF3	0.864			
EFFC	EFFC1	0.916	0.832	0.898	0.747
	EFFC2	0.902			
	EFFC3	0.768			
INT	INT1	0.958	0.939	0.961	0.891
	INT2	0.945			
	INT3	0.928			
SN	SN1	0.887	0.903	0.939	0.838
	SN2	0.934			
	SN3	0.924			
SP	SP1	0.806	0.807	0.861	0.554
	SP2	0.780			
	SP3	0.757			
	SP4	0.720			
	SP5	0.649			
SV	SV1	0.815	0.798	0.868	0.624
	SV2	0.690			
	SV3	0.788			
	SV4	0.856			

Note: ATT—Attitude; BENEFIT—Perceived benefits; CBENF—Community benefits; EFFC—Self-efficacy; IBENF—Individual benefits; INT—Behavioral intention; SN—Subjective norms; SP—Perceived susceptibility; SV—Perceived severity.

Table 2 presents the measurement model evaluation of the higher- and lower-order constructs for a reflective-reflective type HOC. The Cronbach’s *α*, CR, outer loadings, and AVE values have met the recommended values [49].

**Table 2:** Measurement model evaluation of higher- and lower-order constructs.

**Higher-order Constructs (HOCs)**					
HOC	LOC	Outer Loadings	Cronbach’s *α*	CR	AVE
BARRIERS	ABR	0.738	0.601	0.786	0.559
	REGOBR	0.901			
	RELGBR	0.565			
CA	MATTN	0.879	0.626	0.842	0.727
	IDISC	0.825			
TRUST	GOV	0.912	0.883	0.925	0.805
	INFO	0.854			
	VAC	0.925			
**Lower-order Constructs (LOCs)**					
LOC	Indicators	Outer Loadings	Cronbach’s *α*	CR	AVE
ABR	ABR1	0.845	0.701	0.830	0.621
	ABR2	0.674			
	ABR3	0.834			
REGOBR	REGOBR1	0.796	0.805	0.873	0.633
	REGOBR2	0.791			
	REGOBR3	0.714			
	REGOBR4	0.874			
RELGBR	RELGBR1	0.922	0.797	0.907	0.831
	RELGBR2	0.901			
MATTN	CA2	0.702	0.775	0.855	0.598
	CA3	0.830			
	CA4	0.851			
	CA5	0.696			
IDISC	CA6	0.793	0.744	0.854	0.661
	CA7	0.806			
	CA8	0.840			
GOV	GOVTRUST1	0.909	0.963	0.971	0.871
	GOVTRUST2	0.939			
	GOVTRUST3	0.956			
	GOVTRUST4	0.910			
	GOVTRUST5	0.952			
INFO	INFOTRUST1	0.901	0.815	0.890	0.730
	INFOTRUST2	0.757			
	INFOTRUST3	0.898			
VAC	VACTRUST1	0.925	0.958	0.969	0.888
	VACTRUST2	0.963			
	VACTRUST3	0.938			
	VACTRUST4	0.943			

Note: BARRIERS—Perceived barriers; CA—Cues to action, TRUST—Trust; ABR—Access barriers; REGOBR—Registration barriers; RELGBR—Religious barriers; MATTN—Media attention; IDISC—Interpersonal discussion; GOV- Trust in the government; INFO—Trust in information sources; VAC—Trust in the vaccination process.

In addition to the reliability and validity measures in Tables 1 and 2, we also need to establish discriminant validity between the constructs. We do this by assessing the heterotrait-monotrait (HTMT) ratio of correlations. Table 3 shows the HTMT results for the non-registered respondents. The correlation estimates for the constructs are below the threshold of 0.90 [50]. Furthermore, the bootstrap confidence intervals do not contain the value of 1, which further supports discriminant validity [50]. We do not consider discriminant validity between the HOCs and LOCs since we used the repeated indicators approach to estimate the HOCs model [49, 51].

**Table 3:** Discriminant validity.

	ABR	ATT	BARRIERS	BENEFIT	CA	EFFC	GOV	IDISC	INFO	INT	MATTN	REGOBR	RELGBR	SN	SP	SV	TRUST
ATT	**0.164** [0.058, 0.239]																
BARRIERS	**1.006** [0.917, 1.110]	**0.333** [0.190, 0.435]															
BENEFIT	**0.212** [0.085, 0.298]	**0.731** [0.614, 0.818]	**0.377** [0.220, 0.484]														
CA	**0.242** [0.153, 0.280]	**0.313** [0.173, 0.466]	**0.268** [0.193, 0.296]	**0.473** [0.317, 0.617]													
EFFC	**0.172** [0.052, 0.222]	**0.385** [0.190, 0.550]	**0.223** [0.102, 0.280]	**0.373** [0.208, 0.518]	**0.420** [0.219, 0.603]												
GOV	**0.192** [0.062, 0.293]	**0.531** [0.359, 0.671]	**0.270** [0.129, 0.401]	**0.644** [0.488, 0.757]	**0.536** [0.380, 0.675]	**0.367** [0.174, 0.541]											
IDISC	**0.171** [0.063, 0.239]	**0.275** [0.120, 0.464]	**0.185** [0.108, 0.200]	**0.426** [0.230, 0.609]	**1.048** [0.976, 1.135]	**0.262** [0.112, 0.439]	**0.384** [0.187, 0.563]										
INFO	**0.162** [0.064, 0.215]	**0.691** [0.491, 0.850]	**0.265** [0.148, 0.350]	**0.660** [0.500, 0.788]	**0.457** [0.265, 0.655]	**0.422** [0.239, 0.603]	**0.732** [0.608, 0.832]	**0.294** [0.130, 0.520]									
INT	**0.203** [0.088, 0.329]	**0.901** [0.836, 0.950]	**0.334** [0.187, 0.458]	**0.737** [0.568, 0.851]	**0.295** [0.175, 0.426]	**0.418** [0.240, 0.577]	**0.482** [0.286, 0.640]	**0.318** [0.155, 0.517]	**0.662** [0.509, 0.796]								
MATTN	**0.241** [0.116, 0.306]	**0.270** [0.126, 0.439]	**0.271** [0.153, 0.343]	**0.401** [0.225, 0.575]	**1.126** [1.065, 1.216]	**0.446** [0.198, 0.658]	**0.530** [0.333, 0.700]	**0.578** [0.362, 0.756]	**0.478** [0.271, 0.679]	**0.209** [0.109, 0.321]							
REGOBR	**0.657** [0.433, 0.837]	**0.227** [0.106, 0.313]	**1.088** [1.032, 1.189]	**0.258** [0.135, 0.367]	**0.217** [0.124, 0.293]	**0.187** [0.059, 0.289]	**0.262** [0.112, 0.457]	**0.141** [0.061, 0.179]	**0.211** [0.108, 0.301]	**0.191** [0.072, 0.282]	**0.226** [0.106, 0.387]						
RELGBR	**0.227** [0.106, 0.313]	**0.434** [0.170, 0.644]	**0.717** [0.600, 0.812]	**0.459** [0.243, 0.663]	**0.155** [0.091, 0.180]	**0.155** [0.052, 0.268]	**0.143** [0.057, 0.259]	**0.115** [0.025, 0.174]	**0.254** [0.086, 0.448]	**0.452** [0.204, 0.649]	**0.151** [0.076, 0.190]	**0.395** [0.209, 0.574]					
SN	**0.220** [0.093, 0.353]	**0.841** [0.750, 0.917]	**0.337** [0.180, 0.467]	**0.820** [0.703, 0.900]	**0.401** [0.238, 0.563]	**0.520** [0.317, 0.679]	**0.539** [0.351, 0.688]	**0.397** [0.222, 0.584]	**0.701** [0.537, 0.832]	**0.885** [0.809, 0.942]	**0.312** [0.143, 0.503]	**0.191** [0.063, 0.279]	**0.443** [0.185, 0.469]				
SP	**0.325** [0.185, 0.469]	**0.512** [0.346, 0.657]	**0.346** [0.248, 0.401]	**0.487** [0.261, 0.671]	**0.381** [0.223, 0.544]	**0.412** [0.257, 0.593]	**0.486** [0.279, 0.659]	**0.263** [0.129, 0.421]	**0.430** [0.259, 0.578]	**0.526** [0.348, 0.676]	**0.385** [0.195, 0.560]	**0.243** [0.125, 0.312]	**0.251** [0.122, 0.340]	**0.566** [0.392, 0.711]			
SV	**0.171** [0.053, 0.237]	**0.533** [0.311, 0.706]	**0.303** [0.175, 0.376]	**0.648** [0.416, 0.802]	**0.436** [0.275, 0.592]	**0.279** [0.107, 0.460]	**0.522** [0.317, 0.671]	**0.373** [0.184, 0.592]	**0.593** [0.349, 0.776]	**0.537** [0.296, 0.713]	**0.384** [0.217, 0.539]	**0.206** [0.104, 0.256]	**0.369** [0.155, 0.605]	**0.569** [0.370, 0.729]	**0.619** [0.433, 0.779]		
TRUST	**0.217** [0.086, 0.298]	**0.708** [0.556, 0.811]	**0.349** [0.204, 0.479]	**0.756** [0.629, 0.842]	**0.500** [0.349, 0.638]	**0.422** [0.252, 0.574]	**0.954** [0.921, 0.979]	**0.360** [0.191, 0.544]	**0.960** [0.906, 1.015]	**0.672** [0.517, 0.786]	**0.494** [0.310, 0.661]	**0.303** [0.162, 0.469]	**0.288** [0.154, 0.475]	**0.724** [0.594, 0.819]	**0.494** [0.297, 0.653]	**0.603** [0.382, 0.756]	
VAC	**0.228** [0.083, 0.365]	**0.765** [0.611, 0.868]	**0.415** [0.228, 0.567]	**0.771** [0.650, 0.857]	**0.360** [0.214, 0.512]	**0.386** [0.182, 0.563]	**0.754** [0.624, 0.842]	**0.282** [0.130, 0.469]	**0.840** [0.719, 0.926]	**0.749** [0.606, 0.848]	**0.337** [0.168, 0.496]	**0.337** [0.161, 0.528]	**0.418** [0.176, 0.623]	**0.790** [0.665, 0.882]	**0.424** [0.220, 0.595]	**0.563** [0.333, 0.733]	**0.955** [0.920, 0.980]

Note: ATT—Attitude; BARRIERS—Perceived barriers; BENEFIT—Perceived benefits; CA—Cues to action, EFFC—Self-efficacy; GOV- Trust in the government; IDISC—Interpersonal discussion; INFO—Trust in information sources; INT—Intention; MATTN—Media attention; REGOBR—Registration barriers; RELGBR—Religious barriers; SN—Subjective norms; SP—Perceived susceptibility; SV—Perceived severity; TRUST—Trust; VAC—Trust in the vaccination process.

To identify any potential unobserved heterogeneity issues, we ran the FIMIX-PLS procedure. This procedure generates model selection criteria that allow researchers to conclude whether unobserved heterogeneity affects the data [52, 53]. As the results of Akaike’s information criterion with factor 3 (AIC_3_) and consistent AIC (CAIC) produce different results (see S2 Appendix), it is an indication of the absence of unobserved heterogeneity issues [54].

### Structural model evaluation

**Fig 2:**
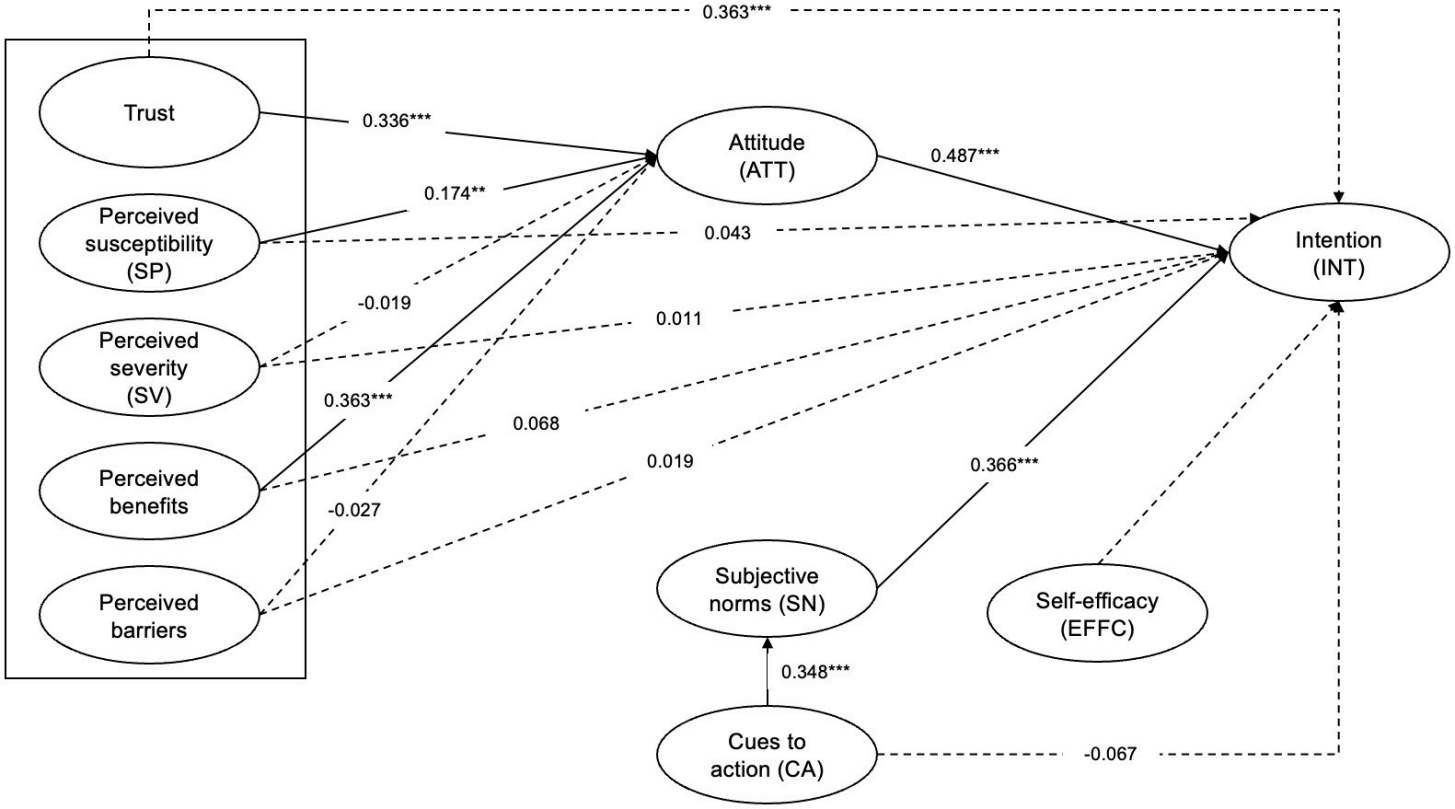
Structural model results on the intention to get vaccinated. Note: (i) *** p < 0.01; ** p < 0.05. (ii) The solid lines represent the significant path coefficients whereas the dotted lines represent the insignificant path coefficients.

Table 4 and Fig 2 present the results of the structural model evaluation. A p-value of less than 0.01, 0.05 and 0.10 implies that the path relationship is significant. Both constructs from the TRA, attitude and subjective norms, were significant in determining vaccination intention. Additionally, perceived benefits, perceived susceptibility, and trust influenced attitude towards vaccination. Cues to action positively influence subjective norms.

**Table 4:** Structural model results for those not registered for COVID-19 vaccination.

	Not Registered (n = 117)	
	Path Coefficients	p-values
ATT -> INT	0.487	0.000***
BARRIERS -> ATT	-0.027	0.709
BARRIERS -> INT	0.019	0.661
BENEFIT -> ATT	0.363	0.000***
BENEFIT -> INT	0.068	0.501
CA -> INT	-0.067	0.225
CA -> SN	0.348	0.000***
EFFC -> INT	0.023	0.661
SN -> INT	0.366	0.001***
SP -> ATT	0.174	0.050**
SP -> INT	0.043	0.507
SV -> ATT	-0.019	0.826
SV -> INT	0.011	0.857
TRUST -> ATT	0.336	0.001***
TRUST -> INT	0.022	0.795

Note:

1. ***p < 0.01, ** p < 0.05.

2. ATT—Attitude; BARRIERS—Perceived barriers; BENEFIT—Perceived benefits; CA—Cues to action; EFFC—Self-efficacy; INT—Intention; SN—Subjective norms; SP—Perceived susceptibility; SV—Perceived severity; TRUST—Trust.

In Table 5, we show the indirect effects of the variables. Attitude towards vaccination mediated three relationships with vaccination intention: perceived benefits, trust and perceived susceptibility. On the other hand, the relationship between cues to action and vaccination intention was also found to be mediated by subjective norms.

**Table 5:** Indirect effects.

	Not Registered (n = 117)	
	Path Coefficients	p-values
BARRIERS -> ATT -> INT	-0.013	0.715
SV -> ATT -> INT	-0.009	0.830
CA -> SN -> INT	0.128	0.013**
SP -> ATT -> INT	0.085	0.076*
BENEFIT -> ATT -> INT	0.177	0.000***
TRUST -> ATT -> INT	0.163	0.008**

Note:

1. ***p < 0.01; ** p < 0.05; * p < 0.10.

2. ATT—Attitude; BARRIERS—Perceived barriers; BENEFIT—Perceived benefits; CA—Cues to action; INT—Intention; SN—Subjective norms; SP—Perceived susceptibility; TRUST—Trust.

## Discussion

Vaccine hesitancy has the potential to weaken efforts to control vaccine-preventable diseases, and it is still a growing concern [55]. Thus, to promote the uptake of vaccines, it is vital to understand the underlying factors influencing why people are either reluctant or hesitant to be vaccinated. This is relevant and important for health authorities and policymakers to enact effective supportive measures and policies that consider these beliefs and behavioural factors. This study highlights two key significant results: none of the Health Belief Model factors significantly predict vaccination intention for the sample of unregistered respondents. However, subjective norms and attitude are important in mediating the relationships between the Health Belief Model factors and vaccination intention. We elaborate on these findings in turn below.

None of the Health Belief Model constructs directly influence the vaccination intention for the group of unregistered respondents. Neither the perceived benefits and barriers nor the perceived severity and susceptibility of the virus can prompt this particular population segment to want to be vaccinated. On the other hand, in other Malaysian studies that did not differentiate between registered and unregistered respondents, researchers found the Health Belief Model constructs to significantly influence vaccination intention. For example, Wong et al. [7] found that a high perception of benefits and low perceived barriers to receiving the vaccine were the two most significant constructs in predicting vaccination intention, while high perceived susceptibility was associated with increased vaccination intention. Similarly, Mohamed et al. [18] also found strong associations between all the Health Belief Model constructs and vaccination intention. Therefore, the absence of any direct effects between the Health Belief Model constructs and vaccination intention in our study deviates from prior research that largely focused on whole population segments, subsequently illuminating the need for targeted strategies for this population segment of unregistered individuals with different behavioral tendencies.

Although no direct effects exist between the Health Belief Model constructs and vaccination intention, indirect effects are present. Subjective norms mediates the relationship between cues to action and vaccination intention among unregistered respondents. Cues to action captures the strategies and initiatives to spur willingness that may trigger a person to feel the need to get vaccinated by stimuli such as exposure to information from mass media or interpersonal discussions. The information acts as a trigger for people to make health-related decisions. Interestingly, the findings suggest that for unregistered individuals, the mechanism through which cues to action can influence the intention to vaccinate is through subjective norms. In other words, subjective norms triggers cues to action for individuals who may be ready but are still unregistered. These individuals require a nudge from people who are important to them (e.g., family, friends, and peers) and who think they should get vaccinated to translate their readiness into intention. This population segment looks upon these ‘influencers’ or role models in their vaccination decision-making process as a reinforcing effect on boosting vaccination intention. This means that identifying ‘influencers’ who can promote vaccine uptake in the population is the way to ensure behavioural change among individuals who are hesitant or uncertain about being vaccinated. These influencers should be someone close and have often built a trusting relationship with the vaccine-hesitant people, or someone with authority. This finding can guide policymakers and healthcare providers in designing persuasive messages and interventions that could effectively stimulate behavioral change by working with these influencers to improve vaccination intention. This implies that while public campaigns are important in promoting and encouraging vaccination uptake, influencers also play a vital role in enforcing the message within specific social orbits. Influencers can be individuals (e.g., heads of households, educators, public figures or role models) and institutions (e.g., ministries, workplaces, schools, and religious bodies). The most effective influencers could also depend on the cultural authority unique to each society. For example, in a multifaith society such as Malaysia, religious authorities can be important influencers in encouraging or discouraging their congregation from taking vaccines [56, 57]. As Islam is the official religion in Malaysia, with 63.5% of the population professing this faith [58], statements released by the Department of Islamic Development at the national level and the respective Islamic Councils at the state level can influence Muslims’ perceptions and subsequent behavior [59, 60]. Therefore, religious authorities should be given adequate information and knowledge on the virus and the importance of getting vaccinated to protect themselves, their families and their congregation from the adverse impact of this infectious pandemic.

Trust, perceived susceptibility and perceived benefits indirectly influence an individual’s vaccination intention through attitude—an indication that an individual’s attitude is integral in influencing behavioural change [39, 61]. Based on this finding, more creative approaches using various modalities are needed to shape people’s attitudes that determine how they react to opportunities and challenges they are faced with. Attitude shifts lie in proper communication through valid information sources, potentially building confidence and trust in vaccines and the vaccination process amongst the unregistered. This is supported by prior studies that have suggested that public health campaigns targeted at promoting vaccine uptake should emphasize educating vaccine-hesitant individuals by conveying clear information pertaining to vaccines and the virus [62–64]. It is common for individuals to assess the risks of contracting the virus based on their knowledge level [65], which is greatly influenced by the media, scientific evidence, community and the government. Without proper communication channels, individuals may opt for alternative therapy, which is not scientifically proven as a cure for COVID-19. Consequently, communication of vaccine-related information or news must be conveyed through official channels to allay any misconceptions the unregistered have regarding vaccines and the vaccination process. Further, the information communicated, particularly scientific messages, must be simple, straightforward and communicated in different vernacular languages of the communities. The proposed measures would help promote confidence in the effectiveness and safety of the vaccine, leading to more positive attitudes and greater vaccination intention.

This study has several limitations. First, data collection was administered using a self-administered online panel skewed towards the more urban population. Hence, the findings may limit generalizability to the overall population. Nevertheless, the data includes participants from less urbanized states such as Kelantan, Sabah, Sarawak and Terengganu, thus increasing the representation of the population. Future studies should seek to obtain equally representative sample from the rural areas. Second, this study employed a cross-sectional design where an individual’s perception of their vaccination intention was obtained at a single point. Future research could consider a longitudinal study which can provide long-term insights into the changes in perceptions on vaccination intention.

## Conclusion

Vaccine hesitancy has gained global recognition as one of the top threats to global health, as it risks undermining efforts to control vaccine-preventable diseases successfully [66]. As countries transition into the endemic phase, vaccine hesitancy remains a valid concern, especially when considering the need for vaccine boosters.

This study examined how factors related to behaviour can illuminate the reasons for vaccine refusal or acceptance and shape and improve health interventions, particularly among the vaccine-hesitant group in a developing country. Although none of the Health Belief Model factors have a direct effect on vaccination intention, the findings revealed that subjective norms and attitude are key mediating factors that could indirectly influence vaccination intention among unregistered individuals. As such, policymakers and key stakeholders can develop effective strategies or interventions to encourage vaccination amongst the unvaccinated for future health pandemics by targeting subjective norms and attitude.

## Supporting information

S1 Appendix List of items. (DOCX)

S2 Appendix FIMIX results. (DOCX)
