# Supplementary material for: COVID-19 vaccine hesitancy and its drivers: An empirical study of the vaccine hesitant group in Malaysia
Source: PLoS One. 2023 Mar 15;18(3):e0282520. doi: 10.1371/journal.pone.0282520 (PMC10016727; doi:10.1371/journal.pone.0282520)
Supplement: S1 Appendix — (DOCX) [file pone.0282520.s001.docx]

**S1 List of items.**

| **Construct** | **Label** | **Indicators** | **Source** |
| --- | --- | --- | --- |
| Perceived Severity | SV1 | If I get Covid-19, other members in my home will get sick | Chu & Liu (2021); Coe et al., (2012) |
|  | SV2 | A person who contracts Covid-19 will die if not treated |  |
|  | SV3 | I believe that Covid-19 has serious negative consequences |  |
|  | SV4 | I believe that Covid-19 is a severe health problem |  |
| Perceived Susceptibility | SP1 | Covid-19 can happen to many people, including my family, loved ones and friends | Chu & Liu (2021); Coe et al., (2012); Yang (2015) |
|  | SP2 | Covid-19 can happen anytime to anyone, such as a healthy individual |  |
|  | SP3 | I am at risk of getting Covid-19 |  |
|  | SP4 | The chance of me contracting Covid-19 is high |  |
|  | SP5 | It is likely that I will get Covid-19 |  |
| Perceived Barriers |  | **Access/Logistics Barriers** |  |
|  | ABR1 | It is inconvenient to get the Covid-19 vaccine as I have no control over the time and place of vaccination | Coe et al., (2012); Yang (2015) |
|  | ABR2 | There is a shortage of the Covid-19 vaccine |  |
|  | ABR3 | The Covid-19 vaccine is not easily accessible to me |  |
|  |  | **Registration** |  |
|  | REGOBR1 | It is inconvenient for me to register for Covid-19 vaccination via MySejahtera | Self-developed |
|  | REGOBR2 | It is inconvenient for me to register for Covid-19 vaccination via the website |  |
|  | REGOBR3 | It is inconvenient for me to register for Covid-19 vaccination via phone call |  |
|  | REGOBR4 | Overall, it is inconvenient for me to register for Covid-19 vaccination |  |
|  |  | **Religion** |  |
|  | RELGBR1 | My religion prohibits me from getting vaccinated | Self-developed |
|  | RELGBR2 | I believe that the Covid-19 vaccine contains ingredients prohibited by my religion |  |
| Perceived Benefits |  | **Perceived individual benefits:** |  |
|  | IBENF1 | Covid-19 vaccines will work in preventing the disease | Chu & Liu (2021) |
|  | IBENF2 | Covid-19 vaccines will be effective in preventing Covid-19 |  |
|  | IBENF3 | If I get the vaccines, I will be less likely to get Covid-19 |  |
|  |  | **Perceived community benefits:** |  |
|  | CBENF1 | Having myself vaccinated against Covid-19 is beneficial for the health of others in my community | Chu & Liu (2021) |
|  | CBENF2 | Covid-19 vaccines protect the health of my community |  |
|  | CBENF3 | Covid-19 vaccines will enable free travel within and outside of the country |  |
| Cues to Action |  | **In the past 1 year, how much attention have you paid to news related to Covid-19 from:** |  |
|  | CA2 | Television | Yang (2015) |
|  | CA3 | Social media such as Instagram, Facebook, TikTok, Twitter, YouTube, etc. |  |
|  | CA4 | Internet (e.g. news site portals; Google) |  |
|  | CA5 | Radio |  |
|  |  | **In the past 1 year, how often have you discussed Covid-19 in the following contexts?** |  |
|  | CA6 | Family in person | Yang (2015) |
|  | CA7 | Friends in person |  |
|  | CA8 | Family and friends online |  |
| Intention |  | **Please indicate the likelihood that you will:** |  |
|  | INT1 | Try to get vaccinated against Covid-19 | Chu & Liu (2021) |
|  | INT2 | Actually get vaccinated for Covid-19 |  |
|  | INT3 | Get vaccinated if a physician offered you Covid-19 vaccines |  |
| Attitude |  | **Using the following adjective scales, please indicate how much you feel that getting vaccinated for Covid-19 is:** |  |
|  | ATT1 | 1 "Unfavourable" to 5 "Favorable" | Chu & Liu (2021); Yang (2015) |
|  | ATT2 | 1 "Bad" to 5 "Good" |  |
|  | ATT3 | 1 "Harmful" to 5 "Beneficial" |  |
| Subjective norms | SN1 | People who are like me will get vaccinated for Covid-19 | Chu & Liu (2021) |
|  | SN2 | People who are important to me will get vaccinated for Covid-19. |  |
|  | SN3 | People who are important to me think that I should get Covid-19 vaccines |  |
| Self-efficacy | EFFC1 | I know just what to do to get the Covid-19 vaccine | Yang (2015) |
|  | EFFC2 | I know how to get the Covid-19 vaccine |  |
|  | EFFC3 | It is easy for me to get the Covid-19 vaccine if I wanted to |  |
| Trust |  | **Trust in the vaccine process** |  |
|  | VACTRUST1 | When it comes to the Covid-19 vaccine process, how much do you trust the World Health Organization? | Freimuth et al. (2018) |
|  | VACTRUST2 | When it comes to the Covid-19 vaccine process, how much do you trust the National Pharmaceutical Regulatory Agency (NPRA)? |  |
|  | VACTRUST3 | Overall, how much do you trust that the organizations involved in the Covid-19 vaccine make their decisions with the public's best interest in mind? |  |
|  | VACTRUST4 | Overall, how much do you trust that all involved organizations do a good job when it comes to the Covid-19 vaccine? |  |
|  |  | **Trust in information sources** |  |
|  |  | How much do you (trust) or (distrust) each of the following as a source of information about the coronavirus pandemic? |  |
|  | INFOTRUST1 | 1. Mainstream media and news such as TV news, newspapers, radio news | Woko et al. (2020) |
|  | INFOTRUST2 | 2. Social media such as Instagram, Facebook, TikTok, Twitter, YouTube, etc. |  |
|  | INFOTRUST3 | 3. Public health officials or agencies |  |
|  |  | **Government Trust** |  |
|  | GOVTRUST1 | During a major crisis, the government informs you about the concerned crisis. How much trust do you generally have in information provided by the government about Covid-19? | van der Weerd et al. (2011) |
|  | GOVTRUST2 | How much trust do you have in measures already taken by the government against Covid-19? |  |
|  | GOVTRUST3 | How much trust do you have in the government with respect to fighting the Covid-19 pandemic? |  |
|  | GOVTRUST4 | What do you think of the decisiveness of the government in taking safety measures against the Covid-19 in the Malaysia? |  |
|  |  | How much trust do you generally have in the government, irrespective of crisis management? |  |
