# Supplementary material for: COVID-19 vaccine hesitancy and its drivers: An empirical study of the vaccine hesitant group in Malaysia
Source: PLoS One. 2023 Mar 15;18(3):e0282520. doi: 10.1371/journal.pone.0282520 (PMC10016727; doi:10.1371/journal.pone.0282520)
Supplement: S2 Appendix — (DOCX) [file pone.0282520.s002.docx]

**S2 FIMIX results.**

|  | **1** | **2** | **3** |
| --- | --- | --- | --- |
| AIC (Akaike's Information Criterion) | 2206.686 | 2117.501 | **2079.068** |
| AIC3 (Modified AIC with Factor 3) | 2240.686 | 2186.501 | **2183.068** |
| AIC4 (Modified AIC with Factor 4) | 2274.686 | **2255.501** | 2287.068 |
| BIC (Bayesian Information Criteria) | **2300.599** | 2308.091 | 2366.334 |
| CAIC (Consistent AIC) | **2334.599** | 2377.091 | 2470.334 |
| HQ (Hannan Quinn Criterion) | 2244.813 | **2194.878** | 2195.695 |
| MDL5 (Minimum Description Length with Factor 5) | **2948.255** | 3622.451 | 4347.399 |
| LnL (LogLikelihood) | -1069.34 | -989.751 | -935.534 |
| EN (Entropy Statistic (Normed)) |  | 0.807 | 0.859 |
| NFI (Non-Fuzzy Index) |  | 0.851 | 0.87 |
| NEC (Normalized Entropy Criterion) |  | 22.544 | 16.548 |
